# INNOVATIVE VOLUNTEER PROGRAMS TO SUPPORT FAMILY CAREGIVERS AND PERSONS LIVING WITH DEMENTIA

**DOI:** 10.1093/geroni/igad104.1084

**Published:** 2023-12-21

**Authors:** Noelle Fields, Ling Xu, Allison Gibson

## Abstract

There is a continued need for accessible and cost-effective community-based services and supports for family caregivers and persons living with Alzheimer’s disease and related dementia (ADRD). Utilizing trained volunteers may offer a scalable approach to filling gaps in ADRD care and support. This symposium will provide evidence for utilizing trained volunteers in innovative interventions designed for family caregivers and persons living with dementia. The first paper presents findings from the Senior Companion Program Plus, an intervention using lay providers to deliver a culturally informed psychoeducational intervention with African American ADRD family caregivers. The second paper highlights the Faith Care Family Project which utilizes trained church volunteers from predominantly African American churches to reach African American ADRD family caregivers. The third paper explores the PorchLight Project, a dementia-capable training and respite delivered by volunteers for older people living in the community throughout Minnesota and in select regions of North Dakota. The fourth paper presents findings from an intervention using trained, experienced Chinese dementia caregivers to be volunteer mentors to newer dementia caregivers in the same ethnic community. The final paper presents findings from a telephone-based, intergenerational reminiscence intervention with college student volunteers and persons living with cognitive impairment that incorporates digital storytelling. The symposium will conclude with a critical reflection on the empirical contributions needed to advance scholarship on leveraging volunteers to meet the needs of family caregivers and persons living with ADRD.

